# Efficient Phosphate Adsorption by Ball-Milled Fe_3_O_4_–Modified Biochar Derived from Agricultural Waste

**DOI:** 10.1021/acsomega.5c03908

**Published:** 2026-02-12

**Authors:** Xiaoqing Meng, Yu Shen, Lin Wang, Yuqi Song, Cansheng Yuan

**Affiliations:** † 154504 (Jiangsu Open University, Nanjing 210036, China; ‡ 74584 Nanjing Forestry University, Nanjing 210037, China)

## Abstract

Phosphorus is a critical factor contributing to eutrophication in aquatic environments, with agricultural nonpoint source pollution identified as its primary source. The development of efficient, eco-friendly, and renewable adsorbents is of significant importance for controlling phosphorus pollution in rural aquatic bodies. Biochar, a porous carbonaceous material, has demonstrated considerable adsorption potential; yet it exhibits limited affinity for phosphate, necessitating performance enhancements through modification. In this study, biochar was prepared from pig manure and wheat husk via high-temperature pyrolysis, and modified by loading nano-Fe_3_O_4_ using a ball milling process, which offers a greener alternative to chemical treatments. The material’s structure was characterized using Brunauer–Emmett–Teller analysis and scanning electron microscopy. Adsorption experiments, including kinetic modeling, isotherm fitting, pH variation, ion interference, and regeneration assessments, were conducted to investigate phosphate removal performance. The wheat husk-derived biochar modified with iron oxide at 800 °C exhibited a specific surface area of 181.71 m^2^·g^–1^, approximately 420 times greater than that of its unmodified counterpart. It followed a pseudo-second-order kinetic model and Langmuir isotherm, with a maximum phosphate adsorption capacity of 125.38 mg·g^–1^ under an initial concentration of (50 mg·L^–1^, pH 7.0, and 8 h equilibrium time), indicating that chemisorption may play a role in the adsorption process. The material demonstrated optimal performance at neutral pH, with calcium ions showing the greatest inhibitory effect on adsorption. After five adsorption–desorption cycles, the removal efficiency remained above 89%, confirming the material’s robust regeneration capacity. Overall, the ball-milled iron oxide modification provides a sustainable and effective means to enhance biochar functionality. The resulting magnetic biochar exhibits high adsorption efficiency, rapid kinetics, pH sensitivity, and excellent reusability, making it a promising candidate for addressing nonpoint source phosphorus pollution in aquatic systems.

## Graphical abstract

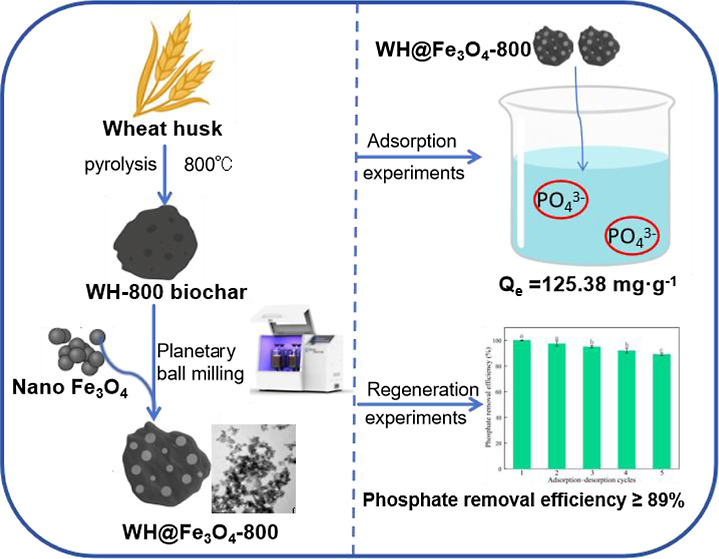

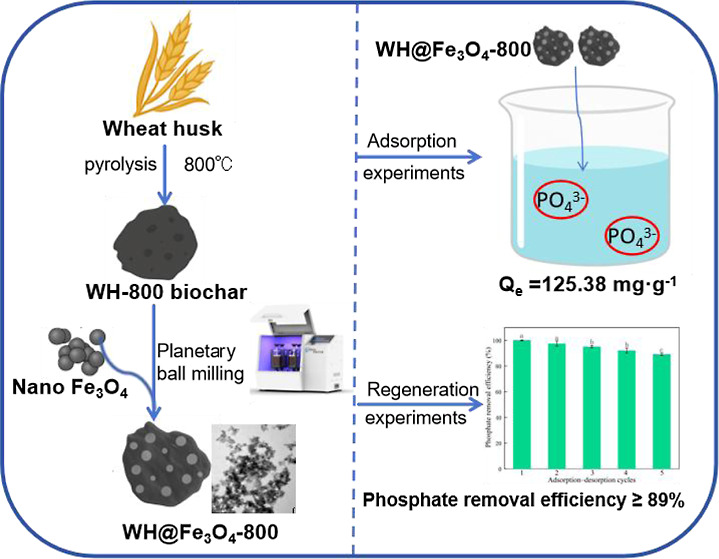

## 1 Introduction

Phosphorus pollution, primarily driven by agricultural nonpoint source runoff, is a key contributor to eutrophication, causing algal blooms, hypoxia, and ecological degradation in aquatic systems.^1–3^ As an effective low-cost adsorbent derived from biomass waste, biochar has gained attention for phosphorus removal due to its porous structure and environmental benefits.^4–6^ Among various materials investigated for phosphorus adsorption, biochar has attracted increasing interest due to its sustainability, tunable surface properties, and low cost.^2,5^ Recent studies have demonstrated the feasibility of using biochar derived from agricultural waste to mitigate phosphorus contamination in rural water systems.^3,6^ However, pristine biochar has a limited affinity for anionic pollutants such as phosphate, particularly under neutral to alkaline conditions, which necessitates effective modification strategies to enhance its adsorption capacity.^7,8^

Magnetite nanoparticles (nano-Fe_3_O_4_) are widely used to functionalize biochar owing to their strong phosphate affinity and magnetic separation capability.^9–12^ However, traditional loading methods such as wet impregnation and sol–gel synthesis often involve complex procedures, nanoparticle agglomeration, and the potential for environmental residues, thereby limiting their large-scale application.^11,14^ In contrast, ball milling presents a green, efficient, and additive-free physical modification approach, facilitating the uniform dispersion and mechanical embedding of nanoparticles within the biochar matrix. This method significantly enhances the development of surface area and pore structure while mitigating the environmental risks associated with chemical modifications.^15,16^ Although ball milling has been applied in material activation, its application in the construction of Fe_3_O_4_-functionalized magnetic biochar systems remains underexplored.^17^ Pig manure (PM) and wheat husk (WH) are two representative agricultural residues with complementary elemental compositions and high availability.^18–20^ PM is nutrient-rich, containing inherent minerals such as Ca and Mg, which are favorable for ion exchange, while WH offers lignocellulosic carbon and silica, contributing to structural stability and porosity during pyrolysis.^10,19^ Their contrasting compositions enable a comparative evaluation of Fe_3_O_4_ modification effects across different biochar matrices. Notably, PM- and WH-derived biochars were prepared and evaluated as independent systems rather than blended feedstocks; therefore, potential synergistic effects were not examined in this work. They were selected as typical biomass sources to develop a biochar modified with nano-Fe_3_O_4_ particles via ball milling, aiming to explore their application potential in phosphate removal and waste valorization. These two residues were chosen because they represent contrasting types of agricultural waste, pig manure being nutrient-rich and mineral-laden, while wheat husk is structurally fibrous and silica-rich, allowing for a broader evaluation of the Fe_3_O_4_ modification via ball milling across diverse biochar matrices and rural organic wastes. While most studies focus on single biomass sources, the use of different waste-derived precursors in this work reflects the practical diversity of rural organic wastes and broadens the scope for biochar-based phosphorus removal materials.^18,21^ Despite progress, the combined use of multiple biomass sources and nano-Fe_3_O_4_ modification via ball milling for phosphate removal remains underexplored.^13^

Based on above, we hypothesize that the incorporation of nano-Fe_3_O_4_ into biochar derived from agricultural waste via ball milling will significantly enhance its phosphate adsorption capacity, surface area, and reusability due to increased active binding sites and improved pore structure. This enhanced material is expected to exhibit strong performance in complex aqueous environments, including tolerance to coexisting ions and regeneration cycles. The adsorption performance and underlying mechanisms for phosphate removal were systematically evaluated. A comprehensive adsorption–desorption cycle was conducted to assess the material’s performance in terms of adsorption kinetics, isotherms, pH sensitivity, coexisting ion interference, and regeneration stability. Furthermore, the relationships between structure and performance were elucidated using scanning electron microscopy (SEM), Brunauer–Emmett–Teller (BET) surface area analysis, and various characterization techniques. This study aims to develop a functional biochar material characterized by high adsorption efficiency, environmental adaptability, and reusability, thereby offering both theoretical insights and practical pathways for the valorization of agricultural waste and the control of phosphate pollution in aquatic environments.

## 2 Materials and Methods

### 2.1 Materials and Reagents

The pig manure utilized in this study was collected from a large-scale livestock farm located in Jiangsu Province, China, and subsequently air-dried prior to use. Wheat husk was procured from a local agricultural market, thoroughly washed with water, and dried at room temperature. The selection of pig manure and wheat husk was guided by their complementary physicochemical characteristics and widespread availability in rural regions of China, aiming to enhance generalizability and material performance diversity.^18,20,21^ Throughout the preparation and adsorption experiments, the two feedstocks were processed separately and were not physically mixed. Nano-Fe_3_O_4_ particles (purity ≥99.5%, particle size ∼50 nm) were acquired from Macklin Biochemical Co., Ltd. (Shanghai, China). All other chemicals, including dipotassium hydrogen phosphate (KH_2_PO_4_, ≥99.0%), hydrochloric acid (HCl, ≥36%), sodium hydroxide (NaOH, ≥96%), and absolute ethanol (C_2_H_5_OH, ≥99.7%), were of analytical grade and supplied by China National Pharmaceutical Group Chemical Reagent Co., Ltd. Deionized water with a conductivity of less than 1 μS·cm^–1^ was employed throughout all experiments.

### 2.2 Preparation of Biochar and Nano-Fe_3_O_4_ Modification via Ball Milling

Air-dried pig manure and wheat husk were ground and sieved to pass through a 100-mesh screen. The resulting powders were placed in sealed ceramic crucibles and subjected to pyrolysis at temperatures of 400 °C, 600 °C, and 800 °C in a muffle furnace (SX2-4-13, Shanghai Yidian Instruments) with a heating rate of 10 °C·min^–1^. Following a holding time of 2 h, the biochar samples were allowed to naturally cool to room temperature. The resulting biochars were thoroughly washed with deionized water to remove residual inorganic impurities, then dried at 105 °C for 24 h, ground, and sieved through a 100-mesh sieve (<150 μm) for storage. The biochar samples were designated as PM-400, PM-600, PM-800, WH-400, WH-600, and WH-800, corresponding to the feedstock and pyrolysis temperature.

Based on initial characterization, four biochar samples (PM-400, PM-600, PM-800, and WH-800) were selected for Fe_3_O_4_ modification using ball milling, following the procedure described by Li et al.^22^ The selected conditions (12 h, 250 rpm, and a 2:1 biochar:nano-Fe_3_O_4_ mass ratio) provide adequate mechanical energy to deagglomerate nano-Fe_3_O_4_ and promote uniform dispersion/embedding on biochar, while avoiding overly harsh milling that may cause excessive pulverization or pore blocking; the 2:1 ratio balances Fe_3_O_4_ loading with pore accessibility and magnetic separability. Specifically, 2.0 g of pretreated biochar and 1.0 g of nano-Fe_3_O_4_ (mass ratio 2:1) were added to a 250 mL PTFE milling jar, along with 100 mL of deionized water and 150 g of agate balls with diameter of 10 mm. The mixture was milled for 12 h at a speed of 250 rpm using a PMQW2 planetary ball mill (Nanda Instrument Co., Ltd., Nanjing, China), with the direction of rotation automatically reversed every 2 h.^22,23^ After milling, the slurry was centrifuged at 4000 rpm for 10 min, dried at 105 °C for 12 h, ground, and sieved. The final Fe_3_O_4_-modified materials were denoted as PM@Fe_3_O_4_-400, PM@Fe_3_O_4_-600, PM@Fe_3_O_4_-800 and WH@Fe_3_O_4_-800.

### 2.3 Biochar Characterization

The specific surface area and pore structure of the biochar samples were determined using a Brunauer–Emmett–Teller (BET) surface area analyzer (ASAP 2460, Micromeritics, USA). The surface morphology was examined using scanning electron microscopy (SEM, Zeiss Sigma 500, Germany). Surface functional groups were identified via Fourier transform infrared spectroscopy (FTIR, Thermo Nicolet iS50, USA), with spectra recorded in the range of 400–4000 cm^–1^. The chemical states of iron and phosphorus species on the biochar surfaces were analyzed by X-ray photoelectron spectroscopy (XPS; Kratos AXIS His, monochromated Al Kα source). Zeta potential was measured over pH 2–11 using a Malvern Zetasizer Nano ZS (UK) to evaluate the surface charge characteristics of the materials.

### 2.4 Adsorption Experiment Design

Batch adsorption experiments were conducted to evaluate the phosphate removal performance of the above biochar samples. All experiments were performed under consistent conditions: an initial pH of 7.0, temperature of 25 °C, and a solution volume of 100 mL. A KH_2_PO_4_ solution was utilized to simulate phosphate-contaminated water.

#### 2.4.1 Adsorption Kinetics

To evaluate the adsorption behavior under different environmental conditions, 0.1 g of adsorbent was added to 100 mL of phosphate solution with an initial concentration of 50 mg·L^–1^ (corresponding to 16.2 mg·L^–1^ P or approximately 0.52 mmol·L^–1^). Samples were collected at predetermined time intervals (1, 2, 4, 6, 8, 12, 18, and 24 h), and the residual phosphate concentration was measured. To avoid any change in the adsorbate-to-adsorbent ratio during sampling, each time point was monitored using a separate flask containing identical dosage and solution volume, and was sampled only once. To gain deeper insight into the rate-limiting steps and underlying mechanisms of phosphate adsorption onto the biochar materials, the experimental data were fitted using pseudo-first-order and pseudo-second-order kinetic models. Both models were fitted using nonlinear regression based directly on the original adsorption data (Qt vs t), rather than linearized transformations, in order to minimize distortion and enable more accurate comparison of fitting parameters and regression coefficients, as shown below

1 $$Pseudo‐first‐order\;model:Q_{t}=Q_{e}-Q_{e}\exp\left(-k_{1}t\right)$$

2 $$Pseudo‐second‐order\;model:Q_{t}=\frac{{k_{2}Q}_{e}^{2}t}{1+{k_{2}Q}_{e}t}$$

where *Q*_t_ and *Q*_e_ (mg·g^–1^) represent the amount of phosphate adsorbed at time *t* and at equilibrium, respectively, and *k*_1_ and *k*_2_ are the corresponding rate constants.

#### 2.4.2 Adsorption Isotherms

To ensure comparability and relevance, the four representative samples (PM-800, WH-800, PM@Fe_3_O_4_-800, WH@Fe_3_O_4_-800) were selected for isotherm studies based on their consistent pyrolysis temperature (800 °C) and distinct material types (pristine vs Fe_3_O_4_-modified). These samples exhibited the most representative adsorption behavior in preliminary tests. To analyze adsorption isotherms, KH_2_PO_4_ solutions with initial phosphate concentrations of 1, 5, 10, 25, 50, 75, 100, and 150 mg·L^–1^ (corresponding to 0.33, 1.6, 3.2, 8.1, 16.2, 24.3, 32.4, and 48.6 mg·L^–1^ as *P* or approximately 0.01–0.48 mmol·L^–1^) were prepared. An amount of 0.1 g of adsorbent was added to each solution and shaken for 18 h at constant temperature. The equilibrium phosphate concentration (Ce) was subsequently measured, and the adsorption capacity (Qe) was calculated accordingly.The listed initial concentrations (C_0_) were only used to generate different *C*_e_ values (not plotted on the *x*-axis). To further investigate the nature of phosphate adsorption and assess the interaction between phosphate ions and the adsorbent surface, the equilibrium data were fitted to the Langmuir and Freundlich isotherm models

3 $$Langmuir\;model:\;Q_{e}=\frac{Q_{\max}.K_{L}{.C}_{e}}{1+K_{L}{.C}_{e}}$$

4 $$Freundlich\;model:\;\mathrm{lg}Q_{e}=\frac{1}{n}\mathrm{lg}C_{e}+\mathrm{lg}K_{F}$$

where *C*_e_ is the equilibrium concentration (mg·L^–1^), *Q*_e_ is the equilibrium adsorption capacity (mg·g^–1^), *Q*_max_ is the maximum monolayer adsorption capacity, and *K*_L_, *K*_F_ (mg g^–1^ (mg L^–1^)^−1/*n*^) and *n* are the fitting constants. Specifically, *K*_L_ is the Langmuir constant associated with binding affinity, *K*_F_ is the Freundlich constant indicating adsorption capacity, and *n* is an empirical parameter representing adsorption intensity and surface heterogeneity.

#### 2.4.3 Effect of pH and Coexisting Ions

The effect of solution pH on phosphate adsorption was evaluated by adjusting the initial pH of the phosphate solution to values of 3, 4, 5, 6, 7, 8, 9, and 10 using hydrochloric acid (HCl) or sodium hydroxide (NaOH). In the experiments involving coexisting ions, sodium ions (Na^+^), calcium ions (Ca^2+^), and chloride ions (Cl^–^) were each added at an initial concentration of 100 mg·L^–1^ to assess their influence on phosphate removal.

#### 2.4.4 Regeneration Experiments

To evaluate the reusability of the adsorbents, saturated samples were desorbed by shaking in 0.1 mol·L^–1^ NaOH solution for 30 min. The materials were then washed, dried at 105 °C, and subjected to five consecutive adsorption–desorption cycles.

#### 2.4.5 Calculation of Adsorption Parameters

The adsorption capacity (*Q*_t_) and phosphate removal efficiency (*R*) were calculated using the following equations

5 $$Q_{t}=\frac{\left(C_{0}-C_{t}\right)\times V}{m}$$

6 $$R=\frac{C_{0}-C_{t}}{C_{0}}\times 100\%$$

where *C*_0_ and *C*_t_ are the initial and residual phosphate concentrations (mg·L^–1^), *V* is the solution volume (L), and *m* is the mass of the adsorbent (g).

#### 2.4.6 Quality Control and Data Processing

All adsorption experiments were conducted in triplicate to ensure the reliability of the data. Phosphate concentrations in the supernatant were determined using the molybdenum blue spectrophotometric method (HJ 670-2013). Data analysis, plotting, model fitting, and statistical testing were performed using Origin 2022 and SPSS 22.0 software. One-way ANOVA followed by Tukey’s post hoc test was used to assess significant differences among groups, with a threshold of *p* < 0.05. Results are reported as mean ± standard deviation (*n* = 3).

## 3 Results and Discussion

### 3.1 Microstructure and Surface Functional Groups of Biochars

To elucidate the influence of surface structure and chemical properties on phosphate adsorption behavior, ten types of biochar samples were characterized in terms of specific surface area (*S*_BET_), average pore diameter, and total pore volume. Representative samples underwent further analysis using scanning electron microscopy (SEM). As shown in Table 1, the nano-Fe_3_O_4_ modification via ball milling significantly enhanced the pore structure of the biochars. Specifically, the *S*_BET_ of WH@Fe_3_O_4_-800 reached 181.71 m^2^·g^–1^, approximately 420.6 times greater than that of the unmodified WH-800 (0.432 m^2^·g^–1^), this dramatic increase in surface area and pore volume directly supports its superior adsorption kinetics (Figure S1, the details are shown in Text S1). The high *S*_BET_ not only provides more active sites for adsorption but also facilitates faster mass transfer. PM@Fe_3_O_4_-800, which also showed enhanced *S*_BET_ (54.36 m^2^·g^–1^), demonstrated better adsorption performance than its unmodified counterpart. These trends confirm that nano-Fe_3_O_4_ modification enhances phosphate adsorption primarily through increased surface accessibility and active site availability. In addition to surface area enhancement, the total pore volume also showed a marked increase; WH@Fe_3_O_4_-800 exhibited a pore volume of 0.228 cm^3^·g^–1^, a 57-fold improvement compared to WH-800 (0.004 cm^3^·g^–1^), and PM@Fe_3_O_4_-800 showed an increase from 0.016 cm^3^·g^–1^ to 0.184 cm^3^·g^–1^. Furthermore, the average pore diameter decreased notably after modification, with WH-800 reducing from 38.750 to 5.024 nm and PM-800 from 18.677 to 13.523 nm, indicating the generation of more developed microporous structures. These structural evolutions are likely attributed to the mechanical embedding of Fe_3_O_4_ nanoparticles onto the biochar surface during ball milling, which may induce microstructural reconstruction and partial unblocking of pore channels. Consequently, more active sites are exposed for subsequent phosphate adsorption.^24,25^ To provide a clearer view of the modifications, a comparative table or visual plot summarizing the changes in *S*_BET_, pore diameter, and pore volume is also recommended.

**Table 1:** Specific Surface Area and Pore Structure Parameters of Different Biochar Materials

Biochar sample	*S*_BET_ (m^2^·g^–1^)	average pore diameter (nm)	pore volume (cm^3^·g^–1^)
PM-400	5.651	19.240	0.027
PM-600	3.015	20.245	0.015
PM-800	3.338	18.677	0.016
PM@Fe_3_O_4_ −400	42.617	13.464	0.143
PM@Fe_3_O_4_-600	58.932	12.930	0.191
PM@Fe_3_O_4_-800	54.359	13.523	0.184
WH-400	1.104	13.265	0.004
WH-600	0.732	22.165	0.004
WH-800	0.432	38.750	0.004
WH@Fe_3_O_4_-800	181.711	5.024	0.228

As shown in Figure 1, the unmodified samples PM-800 and WH-800 displayed blocky or lamellar structures with characterized by relatively large particle sizes and well-defined morphologies. In contrast, the Fe_3_O_4_-modified samples (PM@Fe_3_O_4_-800 and WH@Fe_3_O_4_-800) exhibited significantly smaller particle sizes and transformed into more spherical or aggregated structures, indicating improved dispersibility and surface activity. This morphological transformation suggests that the incorporation of nano-Fe_3_O_4_ during the ball milling process not only enhanced the surface characteristics of the biochar but also facilitated the exposure of active adsorption sites and increased surface energy. These microstructural enhancements establish a favorable foundation for subsequent phosphate adsorption.^5,26^

**Figure 1:**
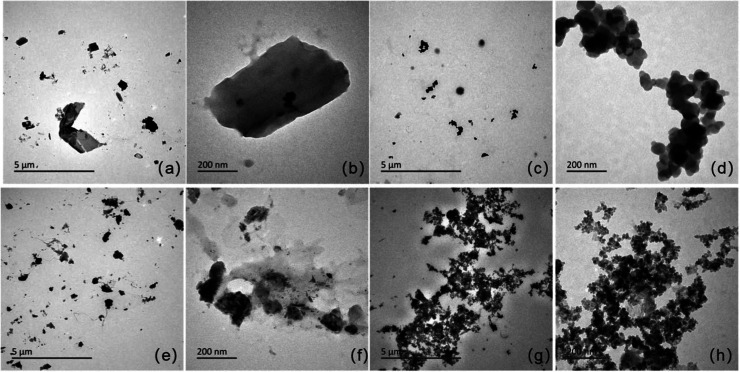
SEM images of various biochar samples. The images include: (a): PM-800, × 8k; (b): PM-800, × 60k; (c): PM@Fe_3_O_4_-800, × 8k; (d): PM@Fe_3_O_4_-800, × 60k; (e): WH-800, × 8k; (f): WH-800, × 60k; (g): WH@Fe_3_O_4_-800, × 8k; (h): WH@Fe_3_O_4_-800, × 60k. Scale bars are shown in all panels (5 μm for (a,c,e,g); 200 nm for (b,d,f,h).

### 3.2 Adsorption Kinetics of Phosphate

To further elucidate the mechanisms governing phosphate adsorption, the experimental data from representative biochar samples were fitted using both pseudo-first-order and pseudo-second-order kinetic models. The nonlinear fitting curves for each model are shown in Figure 2, and the corresponding fitting parameters are summarized in Table 2. Consistent with the graphical results, the pseudo-second-order model provided a better fit than the pseudo-first-order model for all samples, as indicated by higher *R* ^2^ values (0.992–0.996) and lower reduced chi-square values (0.193–0.294).

**Figure 2:**
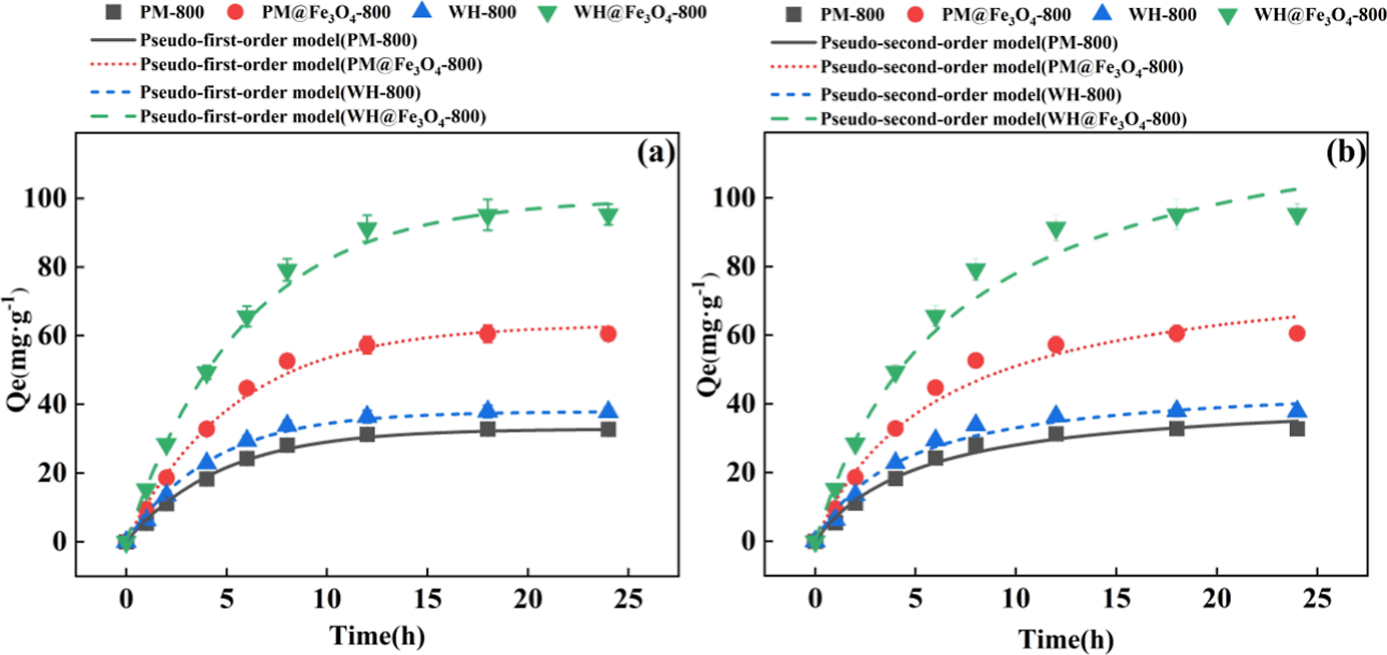
Kinetics fitting curves for phosphate adsorption on four selected biochar samples (PM-800, PM@Fe_3_O_4_-800, WH-800, and WH@Fe_3_O_4_-800) based on nonlinear regression using the pseudo-first-order model (a) and pseudo-second-order model (b). Experimental data are shown with error bars representing standard deviations (*n* = 3).

**Table 2:** Kinetic Model Fitting Equation and Parameters for Phosphate Adsorption by Different Biochar Materials Based on Nonlinear Regression Using the Pseudo-first-order Model (*Q*_*t*_ = *Q*_*e*_-*Q*_*e*_ *exp*(-*k*_1_ *t*)) and Pseudo-second-order Model (*Q*_t_ = $\frac{{k_{2}Q}_{e}^{2}t}{1+{k_{2}Q}_{e}t}$ )

biochar sample	model	equation	*R* ^2^	reduced chi-sqr
PM-800	Pseudo-first-order model	*Q*_*t*_ = 33.65–33.65exp(−0.207*t*)	0.952	0.273
	Pseudo-second-order model	$Q_{t}=\frac{{0.005\times 41.66}^{2}t}{1+\left(0.005\times 41.66\right)t}$	0.996	0494
PM@Fe_3_O_4_-800	Pseudo-first-order model	*Q*_*t*_ = 62.48–62.48exp(−0.199*t*)	0.963	0.154
	Pseudo-second-order model	$Q_{t}=\frac{{0.003\times 78.08}^{2}t}{1+\left(0.003\times 78.08\right)t}$	0.992	0.266
WH-800	Pseudo-first-order model	*Q*_*t*_ = 38.74–38.74exp(−0.228*t*)	0.931	1.253
	Pseudo-second-order model	$Q_{t}=\frac{{0.005\times 47.38}^{2}t}{1+\left(0.005\times 47.38\right)t}$	0.994	0.193
WH@Fe_3_O_4_-800	Pseudo-first-order model	*Q*_*t*_ = 99.2–99.2exp(−0.183*t*)	0.948	0.281
	Pseudo-second-order model	$Q_{t}=\frac{{0.008\times 125.38}^{2}t}{1+\left(0.008\times 125.38\right)t}$	0.995	0.294

This nonlinear regression approach avoids the bias introduced by linearization, thereby offering a more reliable evaluation of model performance.^27^ Importantly, we note that *R* ^2^ alone should not be used as the sole criterion for mechanistic interpretation; rather, the superior performance of the pseudo-second-order model suggests that chemisorption, involving valence forces or electron exchange between phosphate ions and surface functional groups, likely governs the adsorption process.^28^

A closer comparison of the kinetic parameters highlights the substantial improvement brought by Fe_3_O_4_ modification (Table 2). WH@Fe_3_O_4_-800 exhibited the highest equilibrium adsorption capacity (*Q*_e_ = 125.38 mg·g^–1^) and rate constant (*K*_2_ = 0.008 g·mg^–1^·h^–1^), followed by PM@Fe_3_O_4_-800 (*Q*_e_ = 78.08 mg·g^–1^), both of which far outperformed their unmodified counterparts (PM-800 and WH-800, *Q*_e_ = 41.66–47.38 mg·g^–1^). These results are consistent with the structural characterizations in Section 2.1, where nano-Fe_3_O_4_ incorporation greatly enhanced surface area and pore volume, exposing abundant active sites and facilitating rapid ion diffusion. Beyond accelerating adsorption kinetics, the embedded Fe_3_O_4_ nanoparticles also imparted stable magnetic properties to the biochar matrix, ensuring structural integrity during repeated use and simplifying post-treatment separation.^11,12^ Collectively, these findings confirm that the Fe_3_O_4_ modification via ball milling not only improves adsorption efficiency but also enhances the practical applicability of biochar as a recyclable phosphate adsorbent.^29^

### 3.3 Adsorption Isotherms of Phosphate

To further investigate the influence of surface properties on phosphate adsorption performance, four biochar samples (PM-800, WH-800, PM@Fe_3_O_4_-800, and WH@Fe_3_O_4_-800) were subjected to equilibrium adsorption experiments. The adsorption data were analyzed and plotted as Qe–Ce relationships, where Ce denotes the equilibrium concentration. The experimental data were fitted using both Langmuir and Freundlich models through nonlinear regression (Figure 3), and the corresponding fitting parameters are summarized in Table 3. All four samples exhibited typical L-type isotherms, characterized by strong affinity between phosphate ions and surface sites at low concentrations, followed by gradual site saturation at higher concentrations.^30,31^

**Figure 3:**
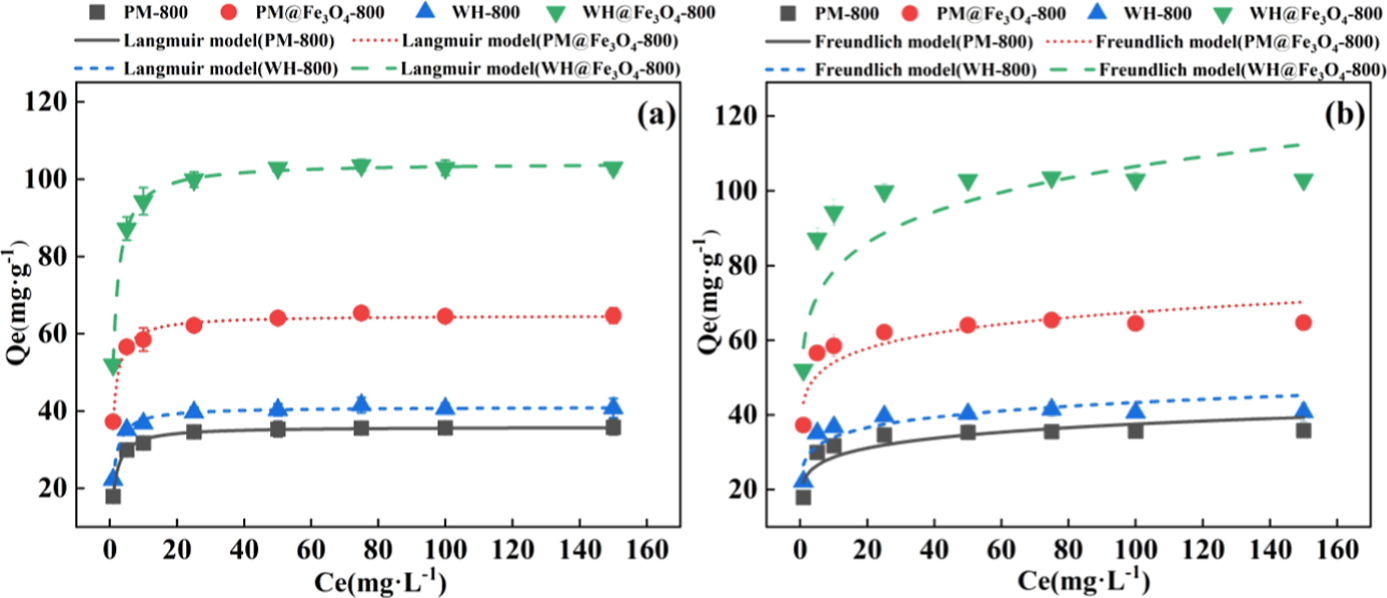
Isotherm fitting curves for phosphate adsorption on four selected biochar samples (PM-800, PM@Fe_3_O_4_-800, WH-800, and WH@Fe_3_O_4_-800) based on nonlinear regression using the Langmuir model (a) and Freundlich model (b). Experimental data are shown with error bars representing standard deviations (*n* = 3). The *x*-axis denotes the equilibrium concentration (Ce, mg·L^–1^).

**Table 3:** Isotherm Fitting Parameters for Phosphate Adsorption by Different Biochar Samples Based on Nonlinear Regression Using the Langmuir and Freundlich Models

biochar sample	Langmuir model				Freundlich model			
	*Q*_max_ (mg·g^–1^)	*K*_L_ (L·mg^–1^)	*R* ^2^	reduced chi-sqr	*K*_F_ (mg·g^–1^·(L·mg^–1^)^^{1/n}^	*n*	*R* ^2^	reduced chi-sqr
PM-800	35.77	0.986	0.996	0.129	21.93	10.03	0.990	5.807
PM@Fe_3_O_4_-800	64.86	1.347	0.997	0.236	43.35	12.02	0.993	13.690
WH-800	40.90	1.175	0.995	0.149	26.67	11.06	0.990	6.686
WH@Fe_3_O_4_-800	104.46	0.993	0.999	0.150	58.01	10.31	0.992	28.855

As shown in Table 3, the phosphate adsorption behavior of all four biochar samples (PM-800, WH-800, PM@Fe_3_O_4_-800, and WH@Fe_3_O_4_-800) fitted better with the Langmuir model, based on nonlinear regression analysis (*R* ^2^ > 0.995 and lower residuals compared to the Freundlich model), suggesting a monolayer adsorption mechanism.^6^ The Fe_3_O_4_ modification via ball milling significantly enhanced the adsorption capacity. Notably, WH@Fe_3_O_4_-800 exhibited the highest theoretical maximum adsorption capacity (*Q*_max_) of 104.46 mg·g^–1^, approximately 2.5 times greater than that of WH-800. Similarly, PM@Fe_3_O_4_-800 achieved a *Q*_max_ of 64.86 mg·g^–1^, about 1.8 times higher than PM-800. Compared with previously reported Fe_3_O_4_-modified wheat husk biochars prepared via wet chemical methods (∼87.3 mg·g^–1^),^32^ WH@Fe_3_O_4_-800 synthesized in this study exhibited significantly higher capacity, underscoring the effectiveness and novelty of the dry ball milling strategy. This solvent-free method avoids hazardous reagents, simplifies processing, and enhances material functionality by increasing surface area, optimizing pore structure, and introducing active Fe–OH and C–O–Fe groups that reinforce phosphate binding.^33^ The Freundlich model further supported these findings: all samples exhibited *n* values >1, confirming the spontaneous nature of adsorption.^34^ Notably, WH@Fe_3_O_4_-800 and PM@Fe_3_O_4_-800 had higher *n* values (10.31 and 12.02), indicating improved affinity and selectivity toward phosphate ions after modification. Taken together, the isotherm results demonstrate that Fe_3_O_4_ incorporation significantly improves both adsorption capacity and surface affinity, with WH@Fe_3_O_4_-800 showing the best overall performance.

### 3.4 FTIR and XPS Analysis of Adsorption Mechanisms

To further confirm the underlying phosphate adsorption mechanism, FTIR and XPS spectra of WH@Fe_3_O_4_-800 before and after phosphate uptake were compared (Figure 4). As shown in Figure 4a, after adsorption, a new absorption band appeared near 1040 cm^–1^, which corresponds to the P–O stretching vibration and evidence the successful binding of phosphate onto the adsorbent surface. Meanwhile, the broad band around 3430 cm^–1^ associated with O–H stretching showed decreased intensity, suggesting the involvement of surface hydroxyl groups in hydrogen bonding or ligand exchange during adsorption.^6^ Additionally, the characteristic Fe–O peak near 580 cm^–1^ showed a slight shift and decrease in intensity, implying an interaction between phosphate species and surface Fe sites.^12^ These spectral changes provide further support for the chemisorption-driven mechanism, aligning well with the results of kinetic and isotherm analyses. It is worth noting that the Fe_3_O_4_ nanoparticles were not chemically grafted but were mechanically embedded into the biochar matrix via the high-energy ball milling process.^26^ This embedding was confirmed through consistent evidence from BET, SEM, and FTIR analyses, reflecting the intimate integration and strong interfacial contact between Fe_3_O_4_ and the carbon framework.

**Figure 4:**
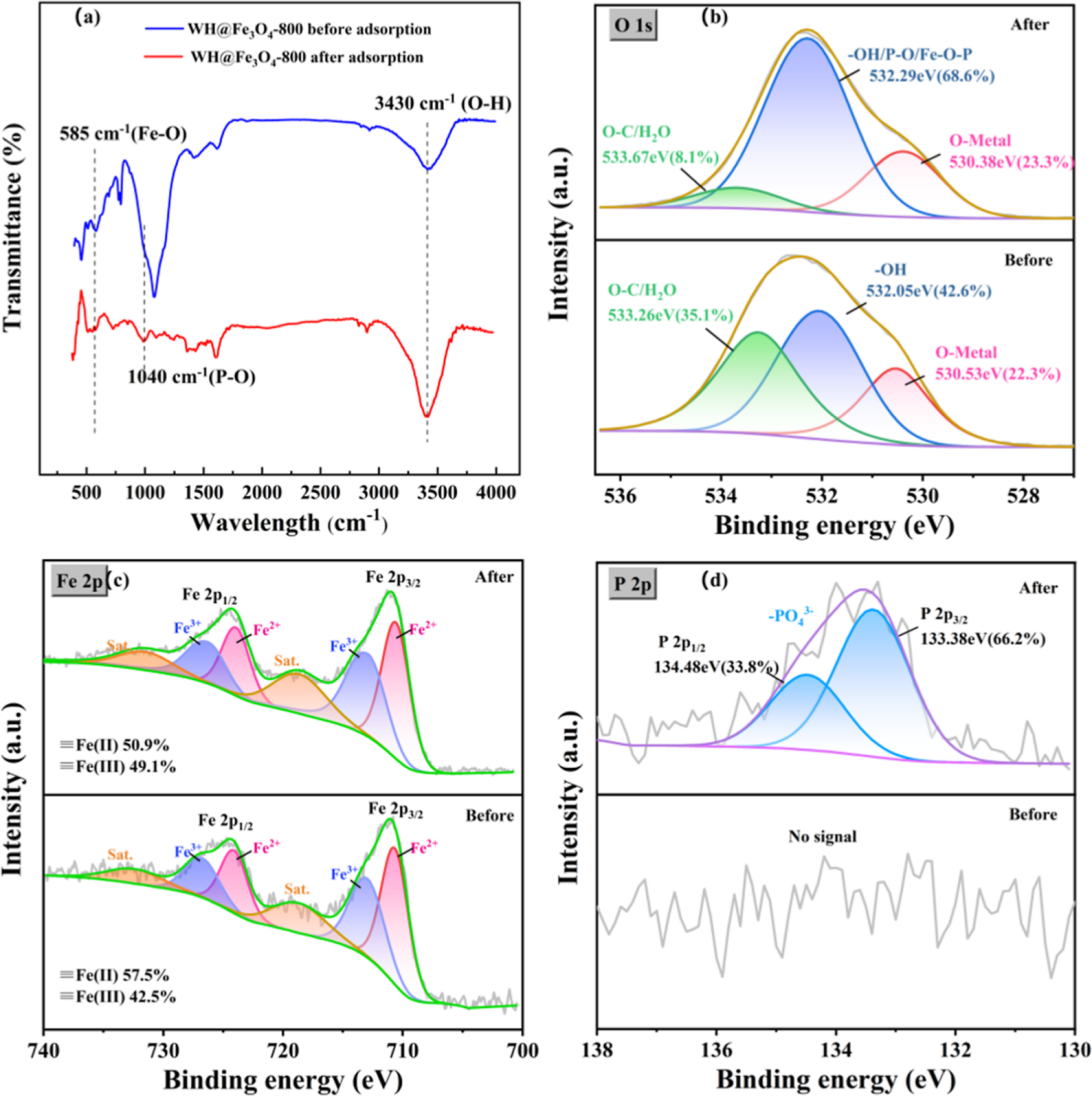
FTIR and XPS spectra of WH@Fe_3_O_4_-800 before and after phosphate adsorption: (a):FTIR spectra; (b) O 1s XPS spectra; (c) Fe 2p XPS spectra; (d): P 2p XPS spectra.

To complement the FTIR observations, XPS spectra of WH@Fe_3_O_4_-800 before and after phosphate adsorption were further analyzed (Figure 4b–d). In the O 1s spectrum, a new component at 532.29 eV corresponding to Fe–O–P appeared, accompanied by a significant decrease in –OH and O–C/H_2_O species, suggesting that surface hydroxyls were replaced by phosphate anions during adsorption.^35^ The Fe 2p spectra revealed an increase in the Fe­(III)/Fe­(II) ratio (from 42.5% to 49.1%), indicative of partial Fe-oxidation and the involvement of Fe active sites.^36^ Most notably, the P 2p spectrum exhibited two distinct peaks at 133.38 and 134.48 eV, assigned to phosphate species bound on the adsorbent surface.^37^ Together with FTIR, these XPS results confirm that phosphate adsorption on WH@Fe_3_O_4_-800 proceeds predominantly via chemisorption involving Fe–O–P bond formation, surface complexation, and hydroxyl substitution. Fe-biochars prepared via conventional wet-impregnation or sol–gel routes often remove phosphate through Fe–OH-mediated ligand exchange/inner-sphere complexation, forming Fe–O–P surface complexes.^32,33^ In contrast, our solvent-free ball-milling strategy immobilizes Fe_3_O_4_ mainly via mechanical embedding, reducing reagent-derived residues and nanoparticle agglomeration while improving interfacial contact and accessible Fe sites,^22,26^ which is consistent with the FTIR/XPS evidence and helps explain the enhanced adsorption performance of WH@Fe_3_O_4_-800.

### 3.5 Effects of Solution pH and Coexisting Ions on Adsorption Performance

Given the superior performance of WH@Fe_3_O_4_-800, this material was selected as a representative to systematically investigate the effects of key environmental parameters, such as initial pH and ion composition, on phosphate removal efficiency, thereby aiding process optimization. As illustrated in Figure 5, the adsorption capacity was significantly influenced by both factors. The phosphate adsorption initially increased with pH and then decreased, reaching its maximum at pH 7.0 (Figure 5a). This trend can be attributed to the combined effect of phosphate speciation and the surface charge of the adsorbent.^38^ Under acidic conditions, H_2_PO_4_^–^ predominates, exhibiting a low negative charge density. Concurrently, the surface of Fe_3_O_4_-modified biochar carries a positive charge at low pH, which enhances electrostatic attraction.^39^ The observed trend is consistent with its point of zero charge (pHpzc = 4.61, Figure S2), below which the surface is positively charged and above which it becomes negative. However, when the pH exceeds 7.0, HPO_4_^2–^ and PO_4_^3–^ became the predominant species, resulting in a negatively charged biochar surface that lead to electrostatic repulsion and a decline in adsorption efficiency. This indicates that WH@Fe_3_O_4_-800 is pH-sensitive and is most suitable for weakly acidic to neutral water environments.

**Figure 5:**
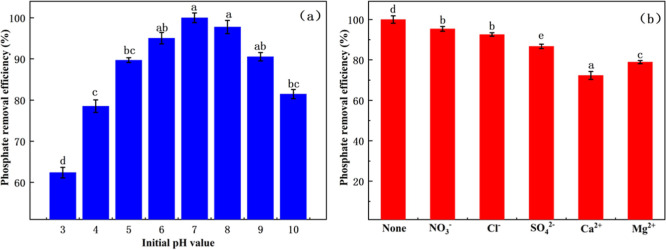
Phosphate removal efficiency of WH@Fe_3_O_4_-800 under different pH values (a) and coexisting ion conditions (b). Each data point represents the mean of three replicates (*n* = 3), and the error bars indicate standard deviations. Different letters above the bars indicate statistically significant differences (*p* < 0.05).

The effect of common coexisting ions on phosphate adsorption was further examined to evaluate environmental applicability. As shown in Figure 5b, all the coexisting ions inhibited phosphate adsorption to varying degrees, with Ca^2+^ exerting the most significant effect, resulting in a 27.7% reduction in adsorption capacity. This phenomenon is likely attributable to the formation of insoluble Ca-phosphate precipitates and the preferential binding of Ca^2+^ to surface functional groups (e.g., –COOH, –OH), thereby occupying active sites.^40^ This unexpected inhibition by Ca^2+^ may stem from its preferential binding with active surface groups, thereby occupying phosphate binding sites, or from competing precipitation processes occurring in solution rather than on the biochar surface, as similarly observed in other studies.^41–43^ Although Na^+^ and Cl^–^ exhibited relatively minor effects, some degree of site competition and charge shielding was nonetheless observed.^44^ In summary, the phosphate adsorption mechanism of WH@Fe_3_O_4_-800 primarily involves electrostatic interaction, surface complexation, and hydrogen bonding. The observed sensitivity to pH and ion species highlights the importance of parameter optimization for practical application scenarios. Its performance is significantly influenced by pH and the presence of coexisting ions, particularly Ca^2+^, underscoring the importance of considering water hardness and potential pretreatment strategies in practical applications. In summary, the phosphate adsorption mechanism of WH@Fe_3_O_4_-800 primarily involves electrostatic interaction, surface complexation, and hydrogen bonding. For comparison, WH-800 was also tested under the same conditions (Figure S3). It showed similar pH- and ion-dependent trends but with markedly lower efficiency, indicating that Fe_3_O_4_ modification enhances adsorption capacity without changing the fundamental mechanism. These findings underscore that environmental parameters, particularly pH and Ca^2+^, remain critical factors for practical applications.

### 3.6 Evaluation of Biochar Reusability

To assess the reusability of WH@Fe_3_O_4_-800, five consecutive adsorption–desorption cycles were performed. As shown in Figure 6, the phosphate removal efficiency reached 100% in the first cycle, but experienced a slight decline in subsequent cycles, decreasing to 89.1% by the fifth cycle. The overall reduction was only 10.9%, with the removal efficiency remaining above 89% across all cycles, which indicates excellent adsorption stability. The sustained performance of WH@Fe_3_O_4_-800 can be attributed to its robust structural stability and strong capacity to regenerate active sites postdesorption. This resilience may stem from its large specific surface area, well-developed pore structure, and the synergistic effects of surface functional groups such as Si–O and C–O.^45,46^ For reference, WH-800 was also evaluated under identical conditions (Figure S4), and although it exhibited a similar declining trend, its overall efficiency was much lower. This comparison further underscores that Fe_3_O_4_ modification not only enhances the initial adsorption capacity but also substantially improves regeneration stability, highlighting the practical advantages of the ball-milled biochar.

**Figure 6:**
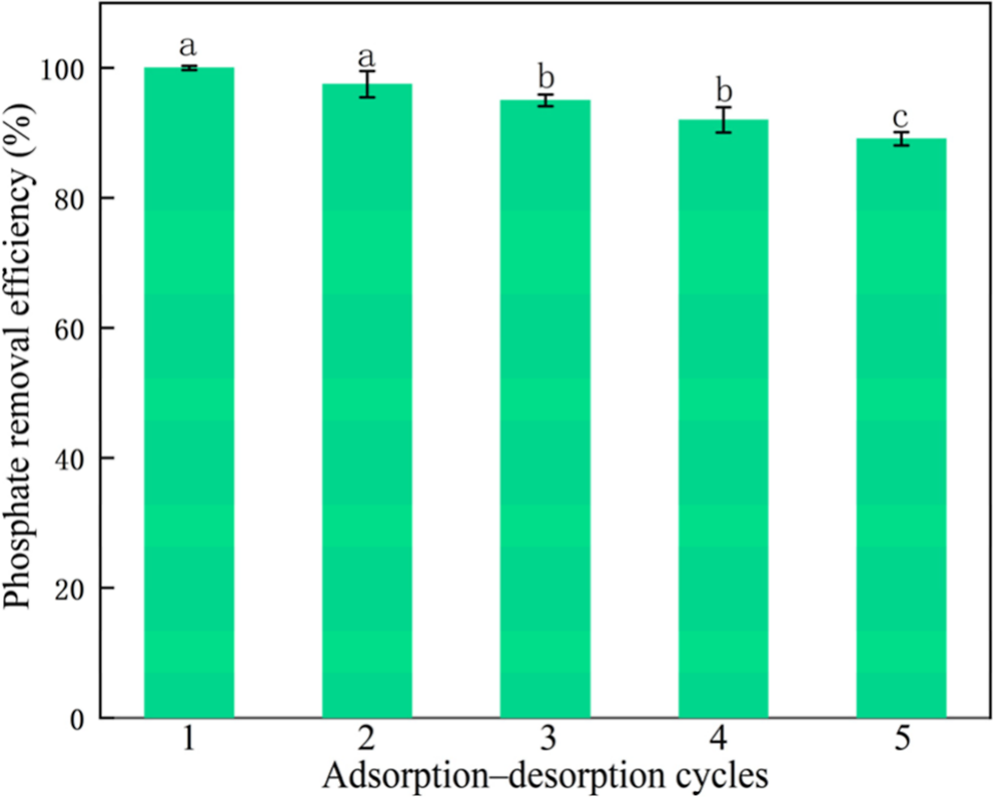
Evaluation of regeneration stability of WH@Fe_3_O_4_-800 for phosphate adsorption. Each data point represents the mean of three replicates (*n* = 3), and the error bars indicate standard deviations. Different letters above the bars indicate statistically significant differences (*p* < 0.05).

In summary, WH@Fe_3_O_4_-800 not only exhibits high initial phosphate adsorption capacity but also maintains strong regeneration performance over multiple cycles, highlighting great potential for practical and sustainable engineering applications. Although WH@Fe_3_O_4_-800 was emphasized due to its superior adsorption capacity, PM@Fe_3_O_4_-800 exhibited similar adsorption kinetics and isotherm trends. Therefore, it is cautiously inferred that PM@Fe_3_O_4_-800 may follow comparable behaviors in terms of pH responsiveness, competitive ion resistance, and regeneration performance, albeit with slightly reduced adsorption efficiency.

## 4 Conclusion

The biochars derived from pig manure and wheat husk, modified with nano-Fe_3_O_4_, exhibited significant advantages in terms of structural characteristics and phosphate adsorption performance. Notably, WH@Fe_3_O_4_-800 demonstrates the most promising results, featuring a specific surface area of 181.71 m^2^·g^–1^, which is over 420 times greater than that of the unmodified WH-800 (0.432 m^2^·g^–1^). This biochar also possesses a more developed pore structure and abundant surface active sites. Kinetic and isotherm model fitting indicated that the adsorption behavior of WH@Fe_3_O_4_-800 followed the pseudo-second-order kinetics and the Langmuir isotherm, with an equilibrium adsorption capacity (*Q*_e_) of 125.38 mg·g^–1^ and a maximum monolayer capacity (*Q*_max_) of 104.27 mg·g^–1^. The Freundlich constant (*n* > 10) further confirmed a chemisorption-dominated process characterized by high affinity and selectivity. Optimal adsorption occurred at pH 7.0, while the material exhibited particular sensitivity to Ca^2+^ interference, suggesting that coexisting ions should be considered in practical applications. Moreover, WH@Fe_3_O_4_-800 maintained a phosphate removal efficiency of 89% after five consecutive adsorption–desorption cycles, indicating excellent stability and reusability. These results suggest that WH@Fe_3_O_4_-800 is a promising candidate for sustainable phosphate remediation in rural water bodies. Nevertheless, the present study was conducted under controlled laboratory conditions, and further validation under complex water matrices is required. In real wastewater, natural organic matter (NOM) and varying ionic strength may introduce additional competition and matrix effects that could influence adsorption and regeneration performance. Future research will focus on evaluating the material’s performance in NOM-containing real wastewater under varying ionic strength and scaling up the synthesis process for practical applications.

## Supplementary Material



## Notes

Additional text, figures and tables, including kinetic adsorption curves of eight biochar samples, zeta potential profiles of WH@Fe_3_O_4_-800 as a function of pH, phosphate removal efficiency of WH-800 under different pH and coexisting ion conditions, and regeneration stability tests of WH-800 (PDF)
