# Supplementary material for: Efficient Phosphate Adsorption by Ball-Milled Fe3O4–Modified Biochar Derived from Agricultural Waste
Source: ACS Omega. 2026 Feb 12;11(7):11094–103. doi: 10.1021/acsomega.5c03908 (PMC12947028; doi:10.1021/acsomega.5c03908)
Supplement: Supplementary file 1 [file ao5c03908_si_001.pdf]

Efficient Phosphate Adsorption by Ball-Milled Fe_3O_4-Modified Biochar Derived from Agricultural Waste

Xiaoqing Meng[1], Yu Shen[2], Lin Wang[1], Yuqi Song[1], Cansheng Yuan[1]*

(1. Jiangsu Open University, Nanjing 210036, China, 2. Nanjing Forestry University, Nanjing 210037, China)

* Corresponding author. College of Rural Revitalization, Nanjing Open University, Nanjing 210036, China. Tel./fax: +86 25 86265120.

E-mail address: pitayameng@163.com

Text S1

To further investigate the effect of pyrolysis temperature and nano-Fe_3O_4 modification on the phosphate adsorption performance of biochars, eight samples were selected for kinetic experiments: PM-400, PM-600, PM-800, PM@Fe_3O_4-800, WH-400, WH-600, WH-800, and WH@Fe_3O_4-800. As illustrated in Figure S1, all samples exhibited a typical two-stage adsorption profile, characterized by a rapid increase in adsorption capacity during the initial phase (0-8 h), which contributed the majority of the total uptake, followed by a slower equilibrium phase. This behavior suggests that phosphate adsorption was initially dominated by surface binding, gradually transitioning to intra-particle diffusion control (Liu et al., 2024). Among the tested samples, WH@Fe_3O_4-800 achieved over 80% of its total adsorption capacity within 8 h and reached equilibrium within 24 h, demonstrating excellent enrichment efficiency and rapid adsorption kinetics. In contrast, unmodified biochars exhibited much lower adsorption rates and capacities, confirming that the superior performance of WH@Fe_3O_4-800 is due to the synergistic effect of biochar–Fe_3O_4 integration.

Reference

Liu C, Yan X, Zhang H X, et al. Biochars and modified-biochars for toxic-metal/metalloid ions sorption in various mixed solution systems: A review on kinetic and isotherm models[J]. Desalination and Water Treatment, 2024: 100404.

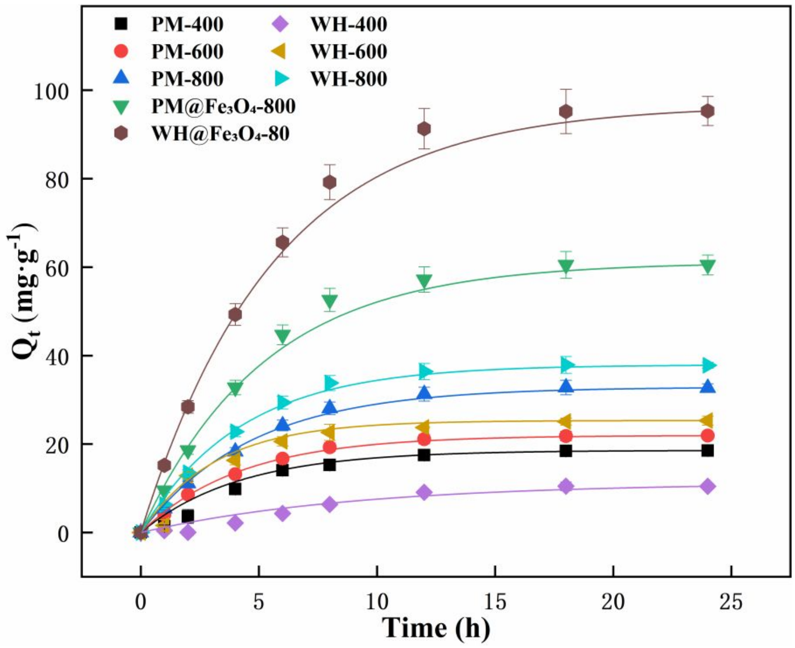

Figure S1 Time-dependent phosphate adsorption performance of different biochars. Experimental data are shown with error bars representing standard deviations (n = 3).

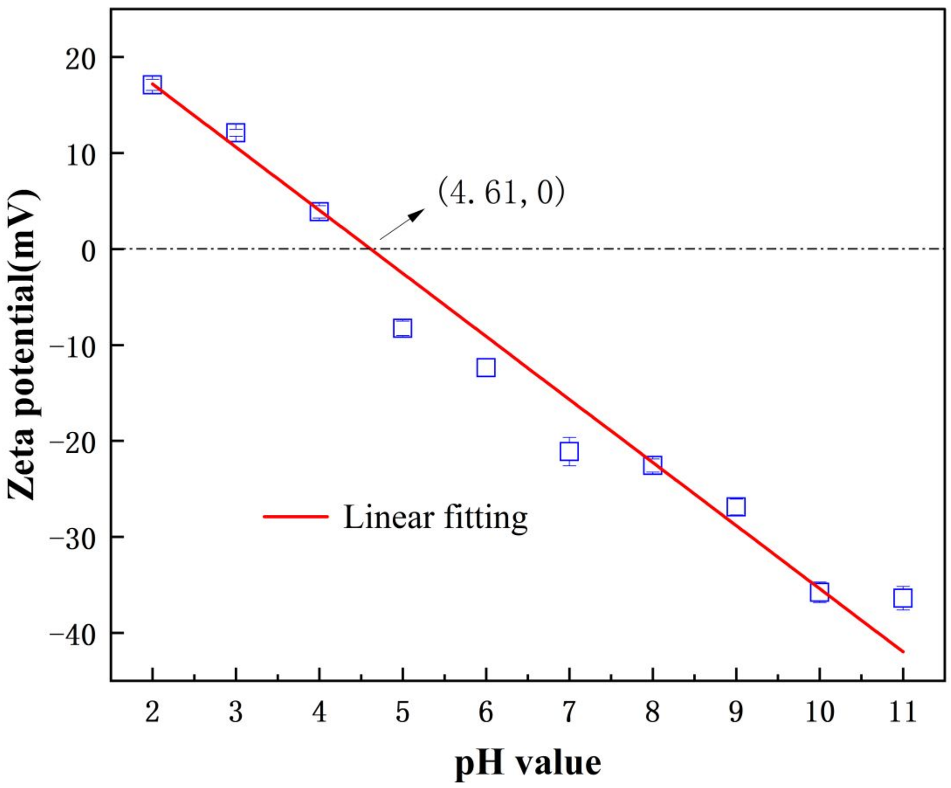

Figure S2 Zeta potential of WH@Fe_3O_4-800 as a function of pH, showing a linear relationship ($Y = -6.573X + 30.33$, $R^2 > 0.98$). The point of zero charge (pHpzc) was determined at 4.61.

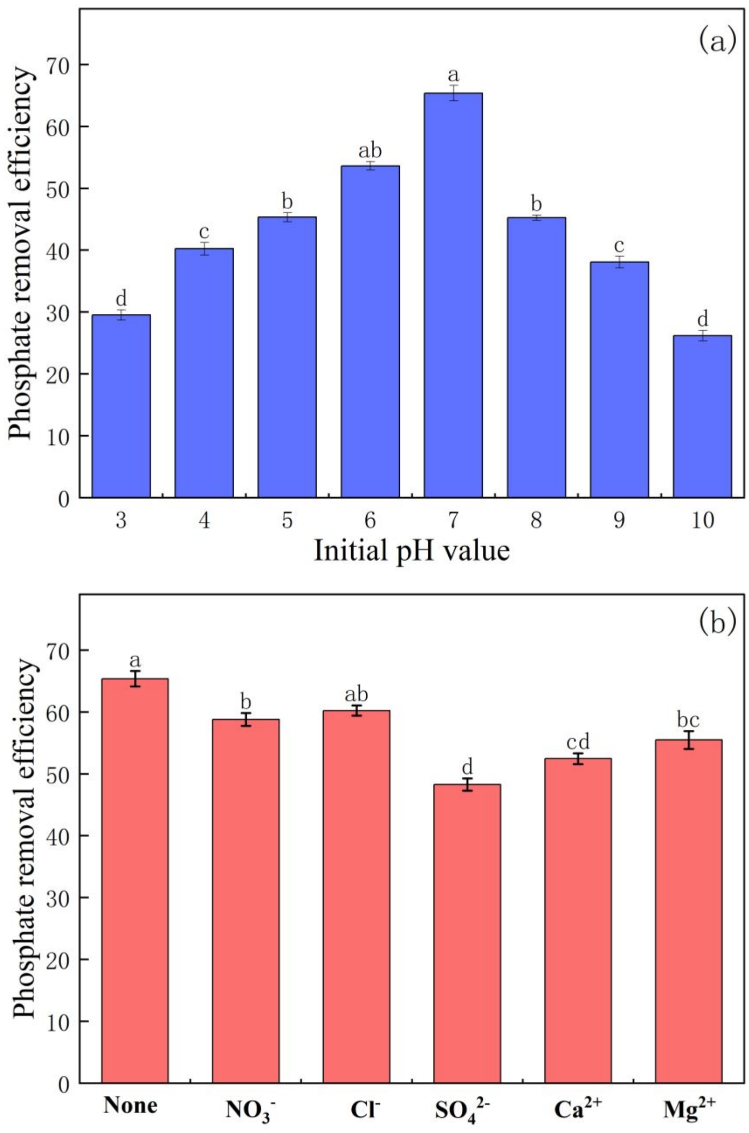

Figure S3 Phosphate removal efficiency of WH-800 under different pH values (a) and coexisting ion conditions (b). Experimental data are shown with error bars representing standard deviations (n = 3). Different letters above the bars indicate statistically significant differences ($p < 0.05$).

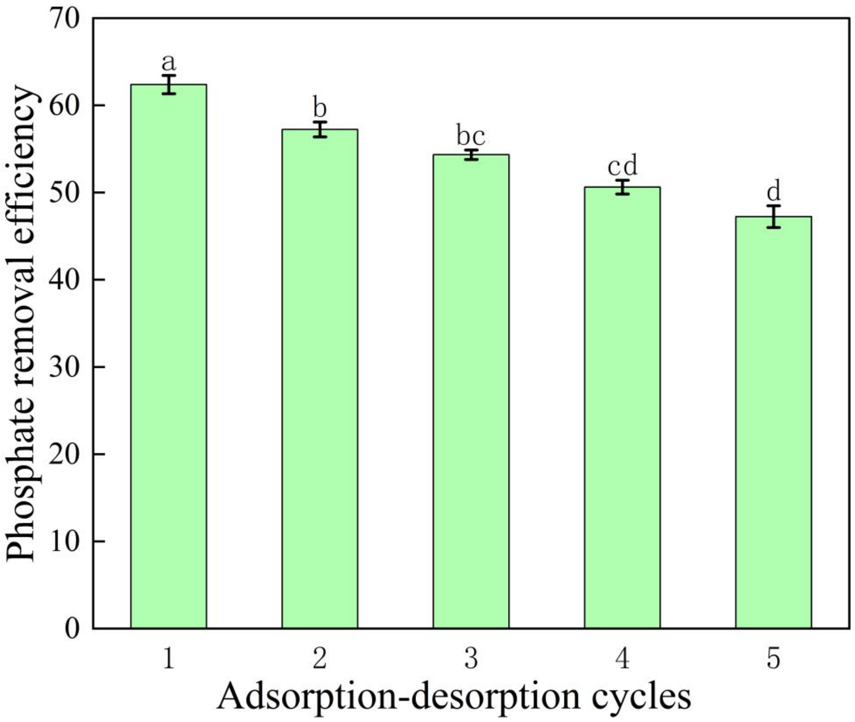

Figure S4 Evaluation of regeneration stability of WH-800 for phosphate adsorption. Experimental data are shown with error bars representing standard deviations (n = 3). Different letters above the bars indicate statistically significant differences ($p < 0.05$).
